# Neutralizing Epitopes and Residues Mediating the Potential Antigenic Drift of the Hemagglutinin-Esterase Protein of Influenza C Virus

**DOI:** 10.3390/v10080417

**Published:** 2018-08-09

**Authors:** Yoko Matsuzaki, Kanetsu Sugawara, Yuki Furuse, Yoshitaka Shimotai, Seiji Hongo, Katsumi Mizuta, Hidekazu Nishimura

**Affiliations:** 1 Department of Infectious Diseases, Yamagata University Faculty of Medicine, Yamagata 990-9585, Japan; ksugawar@med.id.yamagata-u.ac.jp (K.S.); yoshimo@med.id.yamagata-u.ac.jp (Y.S.); shongou@med.id.yamagata-u.ac.jp (S.H.); 2 Institute for Frontier Life and Medical Sciences, Kyoto University, Kyoto 606-8507, Japan; furusey.tohoku@gmail.com; 3 Hakubi Center for Advanced Research, Kyoto University, Kyoto 606-8501, Japan; 4 Department of Microbiology, Yamagata Prefectural Institute of Public Health, Yamagata 990-0031, Japan; mizutak@pref.yamagata.jp; 5 Virus Research Center, Clinical Research Division, Sendai Medical Center, Sendai 983-8520, Japan; hide-nishimura@mte.biglobe.ne.jp

**Keywords:** influenza C virus, escape mutant, antigenic structure, epidemiology

## Abstract

We mapped the hemagglutinin-esterase (HE) antigenic epitopes of the influenza C virus on the three-dimensional (3D) structure of the HE glycoprotein using 246 escape mutants that were selected by a panel of nine anti-HE monoclonal antibodies (MAbs), including seven of the C/Ann Arbor/1/50 virus and two of the C/Yamagata/15/2004 virus. The frequency of variant selection in the presence of anti-HE MAbs was very low, with frequencies ranging from 10^−4.62^ to 10^−7.58^ for the C/Ann Arbor/1/50 virus and from 10^−7.11^ to 10^−9.25^ for the C/Yamagata/15/2004 virus. Sequencing of mutant HE genes revealed 25 amino acid substitutions at 16 positions in three antigenic sites: A-1, A-2, and A-3, and a newly designated Y-1 site. In the 3D structure, the A-1 site was widely located around the receptor-binding site, the A-2 site was near the receptor-destroying enzyme site, and the Y-1 site was located in the loop on the topside of HE. The hemagglutination inhibition reactions of the MAbs with influenza C viruses, circulating between 1947 and 2016, were consistent with the antigenic-site amino acid changes. We also found some amino acid variations in the antigenic site of recently circulating strains with antigenic changes, suggesting that viruses that have the potential to alter antigenicity continue to circulate in humans.

## 1. Introduction

The influenza C virus is a member of the *Orthomyxoviridae* family of enveloped and segmented negative-sense RNA viruses, together with the influenza A virus and influenza B virus. The fourth segment of the influenza C viral genome encodes the hemagglutinin-esterase (HE) glycoprotein, which is present in virions as a homotrimer. The HE monomer, which contains 641 amino acids excluding a signal peptide, is composed of two subunits that are cleaved by a host protease. HE1 (432 amino acids) is the globular region of the HE protein, and HE2 (209 amino acids) is the stalk region [1]. This protein possesses three biological activities: receptor-binding activity for 9-*O*-acetyl-*N*-acetylneuraminic acid, fusion with the host cell membrane, and receptor-destroying activity, which is a neuraminate-*O*-acetylesterase [2,3,4]. HE glycoprotein is the counterpart of both hemagglutinin (HA) and neuraminidase (NA) in influenza A and B viruses. Whereas influenza A virus infects a variety of hosts, influenza C viruses are predominantly found in humans [5]. Influenza C virus usually causes mild upper respiratory tract illness in children but can also cause lower respiratory tract illness, such as bronchitis and pneumonia, particularly in children less than two years old [6,7,8].

The influenza C virus has sporadically been isolated by cell culture; therefore, its epidemiological characteristics are not fully understood. We recently revealed that influenza C viruses can be divided, based on the HE gene, into six genetic and antigenic lineages, designated C/Taylor, C/Mississippi, C/Aichi, C/Yamagata, C/Kanagawa, and C/Sao Paulo [9]. Several lineages cocirculate in a community [10,11], and periodic epidemics of this virus occur as the dominant lineage is replaced every several years in Japan [12]. The antigenicity of the HE glycoprotein is highly stable, and antigenic cross-reactivity occurs between the C/Ann Arbor/1/50 (C/Taylor lineage). The American strain was isolated in 1950, and the Japanese strains were isolated in 2012 (C/Kanagawa lineage and C/Sao Paulo lineage) [12]. Analysis of the evolutionary rate of the HE gene from influenza C viruses, spanning 68 years, also revealed that the rate of 5.20 × 10^−4^ substitutions/site/year for the HE gene is approximately 8–11-fold lower than the evolutionary rate obtained for the HA gene from influenza A (H3N2) viruses and approximately four-fold lower than that obtained for the HA gene from influenza B viruses [9].

One explanation for the antigenic stability of the HE protein is the possible functional constraints on the variation in the immunodominant region [13]. Rosenthal et al. reported the crystal structure of the HE protein and identified the receptor-binding site and the receptor-destroying enzyme site on its structure [14]. They revealed that the HE monomer is composed of three domains: a stem domain, an esterase domain comprising residues 41–150 and residues 311–366, and a receptor-binding domain comprising residues 151–310. However, the immunodominant region of the HE protein has not yet been resolved. We previously obtained a total of 37 monoclonal antibodies (MAbs) specific for the HE of C/Ann Arbor/1/50 virus and identified at least nine antigenic sites (A-1 to A-5 and B-1 to B-4) on the HE protein [15,16,17]. Analysis using eight MAbs with neutralization activity (sites A-1 to A-4) and their escape mutants revealed that 12 different amino acid substitutions identified in a total of 18 mutants were all located at the HE1 subunit, spanning positions 164 to 353 (the 14-amino-acid-long signal peptide is excluded from the numbering) [13]. However, we have not yet placed these epitopes on the three-dimensional (3D) structure of the HE protein.

In this study, to precisely identify the antigenic region of the HE protein, we generated three parent viruses of C/Ann Arbor/1/50 and selected multiple escape mutants of each parent virus. In addition, we selected escape mutants of C/Yamagata/15/2004, a Japanese strain of the C/Yamagata lineage isolated in 2004 [18], using two newly prepared anti-HE MAbs against C/Yamagata/15/2004. Finally, a total of 246 escape mutants were isolated and characterized by their cross-reactions with MAbs. Epitopes identified by changes in the amino acids of the HE molecules were estimated for the first time on the 3D structure of the HE protein. We also analyzed natural isolates obtained until 2016 to investigate the possibility of antigenic drift.

## 2. Materials and Methods 

### 2.1. Viruses

Influenza C viruses, C/Ann Arbor/1/50 and C/Yamagata/15/2004, were propagated in the amniotic cavities of 9-day-old embryonated hen eggs. Three different clones, CJP1, CJP3, and CJP5, and three other clones, CYP1, CYP2, and CYP4, were obtained from well-isolated plaques of Madin–Darby canine kidney (MDCK) cells infected with egg-grown C/Ann Arbor/1/50 and C/Yamagata/15/2004, respectively, which were used as parent viruses. The amino acid sequences of the HE genes of CJP1, CJP3, and CJP5 were identical (Accession no. LC385858), and those of the HE genes of CYP1, CYP2, and CYP4 were also identical (Accession no. LC385859). Subsequently, 25 strains that had been used in our previous studies [5,10,12,15,19,20] and eight strains isolated in Yamagata, Japan in 2016 (C/Yamagata/1/2016, C/Yamagata/4/2016, C/Yamagata/6/2016, C/Yamagata/12/2016, C/Yamagata/24/2016, C/Yamagata/26/2016, C/Yamagata/35/2016, and C/Yamagata/36/2016) were prepared for antigenic analysis. They were all propagated in the amniotic cavities of 8- or 9-day-old embryonated hen eggs and stored at −80 °C until use. For purification of the egg-grown C/Yamagata/15/2004 virus, the amniotic harvests were clarified by centrifugation at 1700× *g* for 10 min at 4 °C, and the virus was pelleted by centrifugation at 120,000× *g* for 60 min at 4 °C. The pellet was suspended in phosphate-buffered saline (PBS) containing 10% glycerol and the quantity of protein in this pellet was measured using the Lowry method.

### 2.2. Antibodies

To select escape mutants in this study, we used eight MAbs (J9, U9, Q5, J14, K16, U1, U2, and D37) of the HE of C/Ann Arbor/1/50, and two MAbs (YA3 and YA5) of the HE of C/Yamagata/15/2004. The eight MAbs against the C/Ann Arbor/1/50 strain were prepared in our previous studies [15,17,21], and the MAbs YA3 and YA5 were produced in this study as previously described [21]. Briefly, six-week-old BALB/c mice were subcutaneously inoculated with a mixture of 100 μg (3.12 × 10^5^ plaque-forming units [PFU]) of purified egg-grown C/Yamagata/15/2004 virus and an equal volume of incomplete Freund adjuvant (DIFCO) three times at intervals of 10–11 days. After 3–5 days of final boosting, the mice were anesthetized and their splenocytes were extracted and fused with myeloma X63-Ag8.653 cells. The hybridoma cells binding to the C/Yamagata/15/2004 whole virus were selected by enzyme-linked immunosorbent assay (ELISA) and then screened using the hemagglutination inhibition (HI) test. The hybridomas producing antibodies with HI activity were cloned twice at terminal dilution and then injected intraperitoneally into pristane-primed BALB/c mice. The resulting ascitic fluids were collected and used as a source of antibodies. The MAbs isotypes were determined using the Ouchterlony double-diffusion method. The animal experiments were carried out with approval from the committee for animal experiments of Yamagata University (project license numbers 22064 and 24026, permitted on March 10, 2010 and March 6, 2012, respectively).

Anti-C/Ann Arbor/1/50 (C/Taylor lineage) chicken antiserum and anti-C/Yamagata/10/89 (C/Yamagata lineage) chicken antiserum were used as previously described. [22]

### 2.3. Enzyme-Linked Immunosorbent Assay

Antibody titers of two MAbs (YA3 and YA5) were determined by ELISA using purified egg-grown C/Yamagata/15/2004 viruses as antigens. Ninety-six-well microplates were coated with 50 μL of 50 μg/mL (1.56 × 10^5^ PFU/mL) viral antigen in 0.05 M carbonate buffer (pH 9.6) and held for 2 h at 4 °C. After blocking with 1% BSA in PBS, the plates were washed four times with PBS containing 0.05% Tween 20 (PBS-T), and 50 μL of two-fold serially diluted MAbs were added to each well. After incubation overnight at 4 °C, the plates were washed four times with PBS-T and incubated for 1 h at room temperature with peroxidase-labeled goat anti-mouse IgG (Invitrogen, Carlsbad, CA, USA). After washing the plates, antibodies were detected by using the Peroxidase Substrate Kit (Bio-Rad Laboratories, Hercules, CA, USA). Optical densities (ODs) were read at 415 nm. ELISA titers are expressed as the reciprocal of the highest antibody dilution with an OD greater than 0.20.

### 2.4. Hemagglutination Inhibition Test

The HI test was performed in 96-well microtiter plates, as previously described [11,23]. Briefly, 50 μL of 8 hemagglutinating units of virus was added to each well containing 50 μL of two-fold serially diluted MAbs or antiserum. After incubation for 30 min at room temperature, 100 μL of 0.5% chicken erythrocytes was added to all wells, and the plates were stored for 60 min at 4 °C. The HI titer was expressed as the reciprocal of the highest antibody dilution that completely inhibited hemagglutination. 

### 2.5. Neutralization Test

The virus neutralization test was performed using 6-well microplates, as previously described [24]. Briefly, a mixture of 2-fold serial dilutions of each MAb and 100 PFU of C/Yamagata/15/2004 were incubated for 30 min at 37 °C and used to inoculate MDCK cells. After 1 h, an agar overlay medium was added, and the cells were incubated at 34 °C for 6 days. Neutralization titers are presented as reciprocals of the highest antibody dilution, causing a >50% reduction in plaque number.

### 2.6. Selection of Escape Mutants

The escape mutants derived from C/Ann Arbor/1/50 were obtained by a plaque method using MDCK cells, as described previously [25], and those derived from C/Yamagata/15/2004 were obtained by an end-point assay using embryonated hen eggs due to their decreased plaque formation ability. Briefly, a 10-fold serial dilution of each parental C/Ann Arbor/1/50 virus (CJP1, CJP3, and CJP5) was mixed with an equal volume of a 1:10 dilution of ascites fluid containing MAb. After incubation for 30 min at room temperature, the mixture was inoculated onto a monolayer of MDCK cells, and an agar overlay medium was added. Ten non-neutralized plaque viruses per experiment were isolated and amplified in MDCK cells. In addition, a mixture of a 10-fold serial dilution of each parental C/Yamagata/15/2004 virus (CYP1, CYP2, and CYP4) and an equal volume of a 1:10 diluted YA3 or YA5 MAb was incubated for 30 min at room temperature and inoculated in the amniotic cavities of 9-day-old embryonated hen eggs. Ten viruses per experiment were isolated from different eggs by the end-point assay. The nucleotide sequence of each mutant HE gene was determined, and the deduced amino acid sequence was compared with that of the respective parental virus.

### 2.7. Nucleotide Sequencing and Phylogenetic Analysis

Viral RNA was extracted from 140 μL culture fluid or amniotic fluid using the QIAamp Viral RNA Mini kit (Qiagen, Hilden, Germany). Synthesis of HE gene cDNA and subsequent polymerase chain reaction (PCR) were carried out in a 25-μL reaction mixture containing 2.5 μL RNA, 0.5μL 20 μM forward primer FluCHEKN8 [9], and 0.5 μL 20 μM reverse primer FluCHER2025 [9] using a One-Step RNA PCR kit (TaKaRa Bio, Kusatsu, Japan). The PCR products were purified with a QIAquick PCR purification kit (Qiagen, Hilden, Germany) and were sequenced using a BigDye Terminator v3.1 cycle sequencing kit (Life Technologies, Carlsbad, CA, USA) and an ABI Prism 3130 sequencer (Applied Biosystems, Foster City, CA, USA). The HE primers for sequencing used in this study were reported in our previous paper [9]. 

The sequences of 8 strains isolated in Yamagata in 2016 (DDBJ/GenBank accession numbers LC385860 to LC385867), together with 107 sequences previously reported [5,9], were analyzed with MEGA6 software (Table S1) [26]. Based on the general time-reversible model, a maximum likelihood phylogenetic tree was constructed with 1000 bootstrapped replicates using the same software.

### 2.8. Structural Analysis

Amino acid positions were plotted on the 3D structure of HE molecules using the PyMOL Molecular Graphics System, version 1.8.6.1 (Schrödinger, LLC, New York City, NY, USA). The crystal structure of HE was obtained from the Protein Data Bank (PDB ID 1FLC).

## 3. Results

### 3.1. Selection of Escape Mutants

The MAbs used for the selection of escape mutants were generated against the HE glycoprotein of the C/Ann Arbor/1/50 strain (eight MAbs: J9, Q5, U9, J14, K16, U1, U2, and D37) [15,16,17] or the C/Yamagata/15/2004 strain (two MAbs: YA3 and YA5). The ELISA titers, HI titers, and neutralization titers of the MAbs are shown in Table 1 and Table 2. Three parent viruses—CJP1, CJP3, and CJP5—derived from C/Ann Arbor/1/50 were neutralized with each MAb, and escape mutants resistant to each MAb (excluding D37) were isolated with a frequency ranging from 10^−4.62^ to 10^−7.58^ (Table 1). D37-resistant mutants could not be isolated. Another three parent viruses—CYP1, CYP2, and CYP4—derived from C/Yamagata/15/2004 were neutralized with each of the MAbs, YA3 and YA5. Escape mutants resistant to each MAb were isolated with a frequency ranging from 10^−7.11^ to 10^−9.25^ (Table 2). Consequently, we isolated 198 and 48 escape mutants with a single amino acid substitution in the HE1 domain of C/Ann Arbor/1/50 (Table 3) and C/Yamagata/15/2004 (Table 4), respectively. By using the parent viruses derived from three different plaques, we were able to obtain various mutants carrying different mutations, as we did in our previous study [24].

### 3.2. Antigenic Sites Revealed by the Reactivities of MAbs with Escape Mutants in HI Tests

The reactivity of each MAb with the panel of escape mutants was examined using the HI tests (Table 3 and Table 4). Based on their reactivities, the seven MAbs against C/Ann Arbor/1/50 were assembled into three groups (A-1, A-2, and A-3) (Table 3), and the MAbs, YA3 and YA5, against C/Yamagata/15/2004 were included in one group, Y-1 (Table 4). 

#### 3.2.1. Site A-1

Single amino acid changes at positions 173, 192, or 269 only affected reactivity with MAb J9, U9, or J14, respectively. Furthermore, a single mutation at positions 125, 172, and 175 selected by MAb J9 affected the reactions of the mutants with MAbs J9 and Q5 or J14, whereas amino acid substitutions at positions 193 and 235 decreased the reactivities of MAbs U9, Q5, and/or J14 with the mutants.

#### 3.2.2. Site A-2

Escape mutants, each possessing a single mutation at positions 65, 68, 351, and 353, were selected by MAb K16, and these mutations affected the reactivity with K16. Moreover, K16 showed decreased reactivity with the other mutants carrying an E193K and a D269N change, which were selected by MAbs belonging to the A-1 site.

#### 3.2.3. Site A-3

A single mutation at positions 164, 198, 201, and 203 affected reactivity with U1 and U2. Interestingly, U1 and U2 showed decreased reactivity with L164P or L201R, but reacted with L164S or L201I.

#### 3.2.4. Site Y-1

Three escape mutants, each possessing a single amino acid change at position 172, 190, or 193, were selected by MAbs YA3 and/or YA5 (Table 4). The reactivities of MAbs YA3 and YA5 with mutants were not affected by amino acid changes at positions 172 and 190, but mutations at positions 172 and 190 affected the reactivity with Q5 and U9, respectively. An amino acid change at position 193 decreased the reactivities of MAbs YA3 and YA5 and also affected the reactivities of MAbs U9, Q5, and J14. Escape mutants with deletions at positions from 192 to 195 and 198 affected reactivity with MAbs YA3 and YA5 and U9, suggesting that these MAbs reacted with the same antigenic site. The epitopes of YA3 and YA5 presumably consisted of residues at positions 192 to 195, and epitope YA3 included residue 198. We designated this antigenic site as Y-1. Interestingly, a deletion at position 198, for which the residue is included in the epitopes U1 and U2 within the A-3 site, did not affect reactivity with U1 and U2, but affected reactivity with U9 and Q5. Therefore, a conformational change due to a deletion at position 198 appears to affect reactivity with MAbs in the Y-1 and A-1 sites.

The reactivity of chicken antisera against C/Ann Arbor/1/50 (Table 3) or C/Yamagata/10/89 (Table 4) with the escape mutants was also examined using the HI test. Escape mutants containing E193K derived from both C/Ann Arbor/1/50 and C/Yamagata/15/2004 showed a four-fold lower reactivity than the respective parent viruses. Reactivity with a deletion mutant at position 198 of C/Yamagata/15/2004 was also reduced. 

### 3.3. Antigenic Structure of Influenza C Virus HE

**Figure 1:**
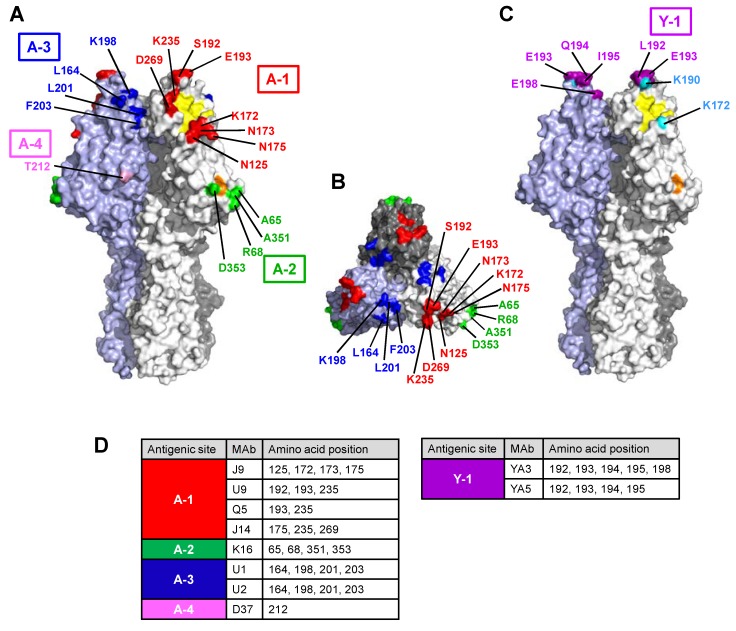
Antigenic sites in the hemagglutinin-esterase (HE) molecule of C/Ann Arbor/1/50 with both (**A**) a side view and (**B**) a top view, and (**C**) C/Yamagata/15/2004. A trimer complex, in which monomers are colored in white, gray, and light blue, is shown in a surface representation with the antigenic sites highlighted: the A-1 site is in red, the A-2 site is in green, the A-3 site is in blue, the A-4 site is in pink, and the Y-1 site is in purple. Residues 172 and 190 discussed in the text are colored light blue in (C). The residues involved in receptor binding and receptor destroying are colored yellow and orange, respectively. Images were created using PyMOL software, and the HE structure was obtained from the Protein Data Bank (PDB ID 1FLC). Amino acid positions are designated by numbering, excluded the 14-amino-acid-long signal peptide. The amino acid at position 212 (pink) in the A-4 site was referred from our previous report [13]. (**D**) Amino acid positions of monoclonal antibody (Mab) binding are shown.

In the 3D structure (Figure 1), the residues of site A-1 were surrounding the receptor-binding site (Figure 1A). Epitope J9, which includes residues 125, 172, 173, and 175, was located near the lower edge, and epitopes U9, Q5, and J14, including residues 235 and 269, were located near the right edge of the receptor-binding site. Residues 192 (epitope U9) and 193 (epitopes U9 and Q5) were located on the top of the HE glycoprotein (Figure 1A). The epitope K16 within the A-2 site, which includes residues 65, 68, 351, and 353, was located near the 9-*O*-acetylesterase active site in the 3D structure (Figure 1A). Residues 164, 198, 201, and 203 within the A-3 site were located on the back side of the A-1 site (Figure 1A,B). Residues 192–195 within the Y-1 site were located on the top of the HE glycoprotein (Figure 1C). 

MAb Q5 recognized the amino acid substitutions in two distantly located epitopes: position 172 (epitope J9) and positions 193 and 235 (epitope Q5). MAb J14 also recognized amino acid substitutions located on epitope J9 (positions 125 and 175) and epitope J14 (positions 235 and 269). Residue 175 is an oligosaccharide attachment site that is used for glycosylation [27], and the escape mutant N175S acquires a new glycosylation site at position 173 (173-NCSNS) instead of position 175. Thus, glycosylation at position 173 appears to influence the reactivity with J14. 

### 3.4. Antigenic Changes in Influenza C Virus Strains Isolated from 1947 to 2016

**Figure 2:**
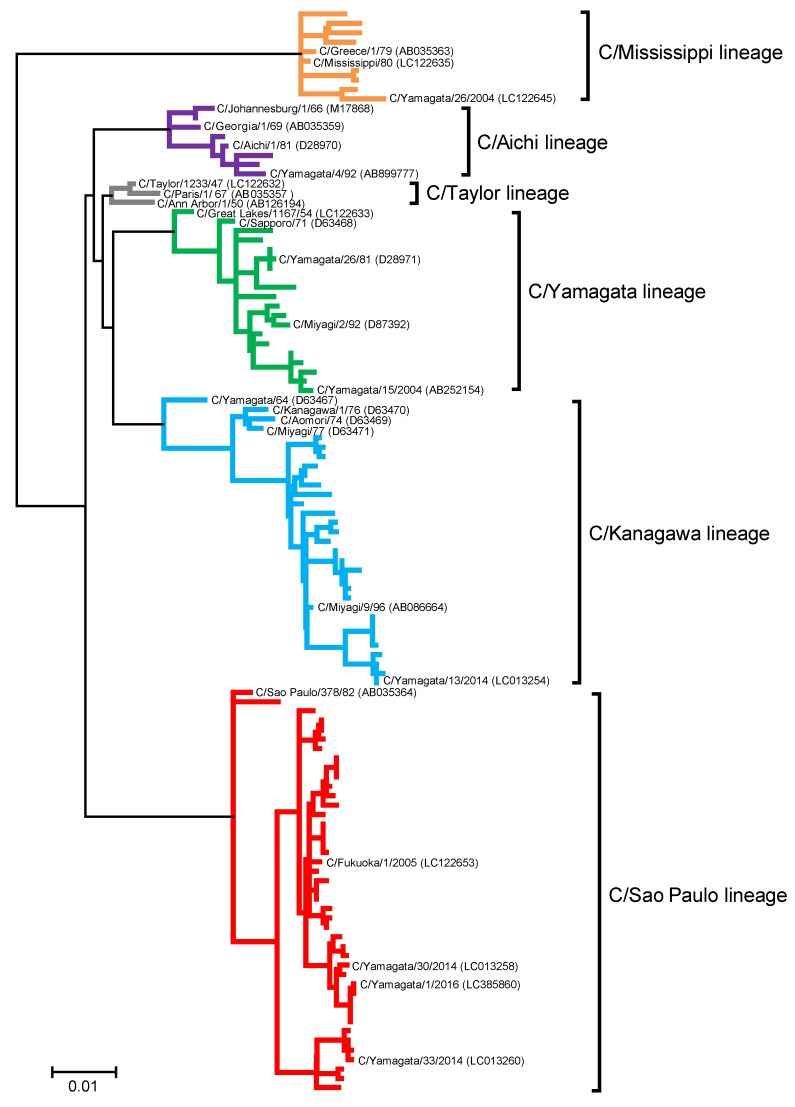
Maximum likelihood phylogenetic tree of the hemagglutinin-esterase (HE) gene of 115 influenza C viruses circulating between 1947 and 2016. The coding region without a signal peptide was used for the analysis, corresponding to nucleotide positions 64 to 1989 of the HE gene. Branches of each genetic lineage are colored orange for the C/Mississippi lineage, purple for the C/Aichi lineage, gray for the C/Taylor lineage, green for the C/Yamagata lineage, blue for the C/Kanagawa lineage, and red for the C/Sao Paulo lineage. Strains that were used for the antigenic analysis presented in Table 5 are shown as names and GenBank/DDBJ accession numbers.

To investigate the possibility of antigenic drift among the six genetic lineages, genetic analyses were performed for influenza C viruses, including 107 strains previously reported between 1947 and 2014 [5,9] and eight strains newly isolated in 2016 (Table S1). These 115 strains were classified into six genetic lineages (Figure 2): 3 in the C/Taylor lineage, 10 in the C/Mississippi lineage, 8 in the C/Aichi lineage, 20 in the C/Yamagata lineage, 31 in the C/Kanagawa lineage, and 43 in the C/Sao Paulo lineage.

**Figure 3:**
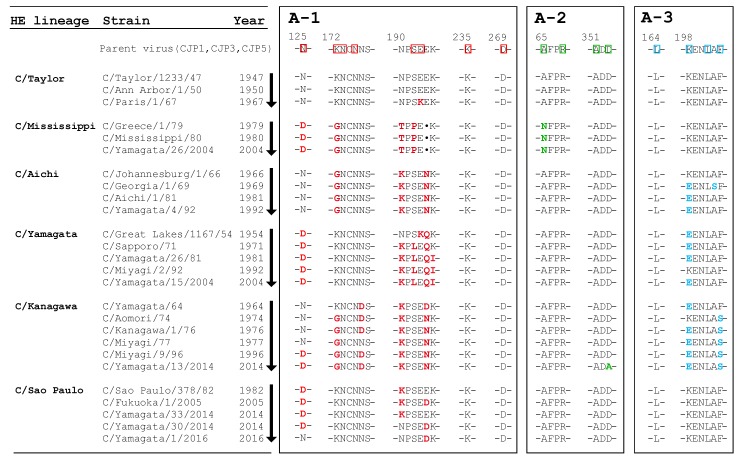
Amino acid sequence alignment of antigenic sites of the influenza C viruses. Boxed residues of the parent virus indicate the antigenic sites A-1 (red), A-2 (green), and A-3 (blue). Amino acid differences from the parent virus are shown in bold letters. The dots indicate deletions. Virus strains correspond to those analyzed in Table 5.

The course of amino acid changes in the antigenic sites for each lineage are shown in Figure 3, and the reactivities of the MAbs with the representative strains are shown in Table 5.

#### 3.4.1. C/Taylor Lineage

There were only three strains belonging to the C/Taylor lineage: C/Taylor/1233/47, C/Ann Arbor/1/50, and C/Paris/1/67. C/Paris/1/67 showed decreased reactivity with U9 and Q5 compared with C/Taylor/1233/47 (Table 5), possibly due to the E193K mutation in the A-1 site (Figure 3).

#### 3.4.2. C/Mississippi Lineage

C/Mississippi/80 lineage strains were sporadically isolated in Japan in two periods: between 1982 and 1985 and between 1992 and 2004 (Figure S1) [5,9,11,18,28,29,30]. Ten viruses belonging to the C/Mississippi lineage were analyzed, and no mutation occurred in the antigenic sites during the period of 1979 to 2004. C/Mississippi lineage strains failed to react with MAbs J9, U9, and Q5 (Table 5), possibly due to the N125D, S192P, and K172G mutations, respectively (Figure 3).

#### 3.4.3. C/Aichi Lineage

We previously reported that C/Aichi lineage strains disappeared from the world after 1992 [9], and eight strains isolated between 1966 and 1992 were analyzed. Amino acid changes were not found in the A-1 and A-2 sites during the 1966–1992 period. The C/Aichi lineage strains failed to react with MAbs J9 and Q5 (Table 5), possibly due to the K172G mutation (Figure 3). Viruses isolated after 1969, including C/Georgia/1/69, C/Aichi/1/81, and C/Yamagata/4/92, had decreased reactivity with U1 and U2 compared with C/Johannesburg/1/66 (Table 5) due to the K198E mutation in the A-3 site (Figure 3).

#### 3.4.4. C/Yamagata Lineage

Viruses belonging to the C/Yamagata lineage circulated predominantly in Japan during the periods from 1988 to 1992 and 1996 to 2000 (Figure S1) [5,9,10,12,31], and no amino acid changes occurred at antigenic sites A-1 and A-2 from 1981 to 2004 when the last strain, C/Yamagata/15/2004, was isolated. There were no strains possessing deletions at positions from 192 to 195 or at position 198 at the Y-1 site. The C/Yamagata lineage strains isolated after 1971 showed decreased reactivity with U9 compared with C/Great Lakes/1167/54 (Table 5), possibly due to the S192L mutation in the A-1 site (Figure 3). Four strains possessing lysine (K) at position 198 in the A-3 site were isolated in Japan during the period of 1991 to 1992 [5]. These strains, such as C/Miyagi/2/92, showed a greater than 10-fold higher reactivity with U1 and U2 than those possessing glutamic acid (E) at position 198 (Table 5). However, the C/Yamagata lineage strains isolated after 1996 again possessed glutamic acid (E) at position 198.

#### 3.4.5. C/Kanagawa Lineage

Viruses belonging to the C/Kanagawa lineage dominantly circulated in Japan between 2002 and 2004 (Figure S1) [12]. We previously reported that C/Kanagawa lineage strains circulating between 2002 and 2004 could be classified into three distinct antigenic groups: a C/Aomori/74-like group, a C/Kanagawa/1/76-like group, and a C/Miyagi/77-like group, with representative strains being C/Aomori/74, C/Kanagawa/1/76, and C/Miyagi/77, respectively [23]. The variable reactivities with MAb U9 and MAbs U1 and U2 among these three strains (Table 5) were possibly due to amino acid substitutions at position 176 and at position 198, respectively (Figure 3) [23]. Subsequently, all strains of the C/Kanagawa lineage during or after 2012 belonged to the C/Kanagawa/1/76-like group, including C/Miyagi/9/96 [23], which acquired the D353A mutation in the A-2 site in 2014 (Figure 3). Moreover, we found that the loss of reactivity with MAb J9 among the C/Kanagawa lineage strains after 1964 (Table 5) occurred due to K172G and/or N125D mutations (Figure 3).

#### 3.4.6. C/Sao Paulo Lineage

Viruses belonging to the C/Sao Paulo lineage dominantly circulated in Japan between 2005 and 2016 (Figure S1) [9,12]. All 2016 strains isolated in Yamagata were grouped in the C/Sao Paulo lineage. C/Sao Paulo lineage strains circulating in or after 2014 showed a distinct reactivity with MAb J9 [20]. Of the eight strains isolated in 2016, three strains failed to react with MAb J9, three strains reacted well with J9, similar to a C/Yamagata/30/2014 strain possessing the K190N mutation, and two strains, such as C/Yamagata/1/2016, showed a 100-fold higher reactivity with J9 than C/Yamagata/30/2014 due to the D125N mutation (Table 5 and Figure 3). According to the 3D structure, residue 190 was far from epitope J9 (Figure 1A,C). However, an escape mutant of the C/Yamagata/15/2004 strain possessing a single mutation of K190N showed an HI titer of 1:640 for reactivity with MAb J9, whereas a parent virus of C/Yamagata/15/2004 demonstrated an HI titer of less than 20. Interestingly, C/Yamagata/30/2014 and C/Yamagata/1/2016, possessing the K190N mutation, failed to react with MAbs YA3 and YA5 in the Y-1 site, similar to the reactivity of C/Great Lakes/1167/54 belonging to the C/Yamagata lineage (Table 5 and Figure 3).

## 4. Discussion

We analyzed the antigenic structure of HEs of C/Ann Arbor/1/50 and C/Yamagata/15/2004 using the respective anti-HE MAbs and their escape mutants. By neutralizing three parent viruses, we were able to obtain more mutants carrying different mutations of the C/Ann Arbor/1/50 strain compared to our previous study [13]. This study contained novel residues at positions 125, 175, 193, and 235 in the A-1 site; at positions 65, 68, and 351 in the A-2 site; and at position 201 in the A-3 site. No escape mutants resistant to MAb D37 were obtained, but a previous study reported that the D37 epitope included the residue at position 212 in the A-4 site [13]. In this study, we found that residue 212 of the A-4 site was far from the residues of sites A-1, A-2, and A-3 in the 3D structure (Figure 1A). In addition, a novel antigenic site Y-1 was defined on the HE protein of the C/Yamagata/15/2004 strain and overlapped with an antigenic site A-1.

A novel finding was that the A-1 site is widely located surrounding the receptor-binding site. In particular, epitope J9, which is composed of residues 125, 172, 173, and 175, was located near the residues forming hydrogen bonds with the HE receptor, which included Y127, T170, G172, Y227, and R292, as reported by Rosenthal et al. [14]. As shown in Figure 3, G172 is found in viruses belonging to the C/Aichi lineage, the C/Mississippi lineage, and the C/Kanagawa lineage, but viruses belonging to the C/Taylor lineage, the C/Yamagata lineage, and the C/Sao Paulo lineage carry K172. As MAb J9 selected several mutants possessing a mutation at position 172, such as K172R, K172Q, and K172N, K172 is a changeable residue presumably involved in antibody recognition.

Before the crystal structure of HE protein was reported, we predicted that residues 172 to 192 would be located close to residue 269 on the HE protein because site A-1 epitopes, recognized by four MAbs (J9, U9, Q5, and J14), overlapped each other [13,16]. However, we found that residues 172 to 175, residue 192, and residues 235 and 269 were distantly located in the 3D structure. Therefore, MAbs Q5 and J14 appear to react with two distantly located regions: on the lower edge of the receptor-binding site (residue 172 and 175), and on the right edge of the receptor-binding site (residue 235 and 269). Decreased reactivity of MAb J14 with the escape mutants carrying the mutation at position 175 may be attributed to a new glycosylation at position 173 by the N175S mutation rather than to a direct interaction. The oligosaccharide chain in this position might hide the J14 epitope from antibody recognition.

Epitope U9 in the A-1 site, which is composed of residues 192, 193, and 235, is located far from epitope J9 because no overlapping reactivity was observed between MAbs J9 and U9 (Table 3). In the 3D structure, epitope U9 is located in the loop on the topside of the HE protein [14]. In this study, the variants with S192L and E193K selected by MAb U9 comprised 37% (43 of 116) of all variants with mutations in the A-1 site (Table 3). Therefore, the loop on the topside of the HE protein may be a key region that can be changed by antigenic recognition. This region may be a major antigenic site in the HE protein of the influenza C virus corresponding to antigenic site B located at the membrane distal end of the H3 HA protein of influenza A virus [32].

In this study, we found that epitope K16 in the A-2 site, which is composed of residues 65, 68, 351, and 353, is near the 9-*O*-acetylesterase active site (S57, D352, and H355) [14]. Although MAb K16 had little hemagglutination inhibition activity, it had high neutralization activity as well as MAbs against the A-1 site (Table 1). Therefore, neutralizing antibodies against the acetylesterase domain of HE protein, such as those against the NA protein of influenza [33], may be generated in humans infected with influenza C virus. In our previous study using various assay systems, we did not obtain evidence that MAb K16 has a higher ability to inhibit receptor-destroying activity than MAbs targeting the A-1 site (J14, J9, and Q5) [34]. Moreover, a mutation at position 353 was suggested to not affect receptor-destroying activity [13]. Residues of the K16 epitope may change while maintaining the receptor-destroying activity of the virus. The biological activities of these escape mutants need to be further evaluated.

Unlike the A-1 and A-2 sites, epitopes U1 and U2 in the A-3 site are far from the biological active site, such as receptor-binding sites or receptor-destroying sites. In the present study, the variants with K198E comprised 49% (29 of 59) of all variants with mutations in the A-3 site (Table 3). An amino acid change of E to K at position 198 was identified in HMV-II cell-grown viruses but not in egg-grown viruses [35]. The E198K mutation may be caused by adaptation of viruses to cell lines. However, variants with the K198E mutation exhibited decreased reactivity with MAbs U1 and U2, which were used for the selection of the variant, suggesting that residue 198 is actually composed of epitopes U1 and U2 in the A-3 site.

A new antigenic site, Y-1, is located in the loop on the topside of the HE protein as well as epitope U9 in the A-1 site. Epitopes YA3 and YA5, which consist of residues at positions 192 to 195 and 198, overlap with epitope U9 in the A-1 site and possibly with epitope Q5 or J14. Interestingly, two escape mutants selected by MAbs YA3 and YA5 possessed deletions at positions 192 to 195 and position 198, respectively, rather than a single amino acid change. Surprisingly, this region may not be essential for viral propagation. Among the natural isolates, a loss of amino acids in the HE protein rarely occurs and only viruses belonging to the C/Mississippi lineage possess a deletion at position 194 (Figure 3). Therefore, this region in the loop, on the topside of the HE protein, seems to be changeable through deletion as well as mutation.

The antigenicity of influenza C virus is highly stable, as indicated by the amino acid sequence identity of more than 93% obtained for the HE1 protein of natural isolates during a 68-year period [9]. Site-by-site selective pressure analysis of the HE gene of the influenza C virus demonstrated that no sites are under significant positive selection [23]. However, amino acids from positions 190 to 195 in the A-1 site, which contain the loop on the topside of the HE protein, were variable among the six distinct antigenic lineages (Figure 3). Moreover, among the six antigenic lineages and within the same antigenic lineage, variations in amino acids were found at residues 125 and 172 (epitope J9), residue 192 (epitope U9), and residues 198 and 203 (epitopes U1 and U2). Therefore, these amino acids are related to the antigenic properties of the lineages of the influenza C virus. We previously reported that amino acids at positions 176 and 198 are associated with the antigenic characteristics of three distinct groups in the C/Kanagawa lineage, the C/Kanagawa/1/76-like group, the C/Aomori/74-like group, and the C/Miyagi/77-like group [23]. As genetic diversity is associated with the effective population size, amino acid variations at positions 176 and 198 seem to be responsible for the dominant circulation of the C/Kanagawa lineage from 2002 to 2004 in Japan. Similarly, among the C/Sao Paulo lineage strains dominantly circulating from 2005 to 2016, several antigenic variants showing different reactivity with MAb J9 were found around 2014 [20] and 2016, and such strains possessed the D125N or K190N mutations (Table 5 and Figure 3). These findings suggest that viruses with potential to alter antigenicity might continue to circulate. Further observations are needed to determine whether future strains of the C/Sao Paulo lineage succeed in the D125N and K190N mutations, which alter reactivity with MAb J9.

The mutations in the antigenic sites of the natural isolates affected the reactivities of the corresponding MAbs in the HI test (Table 5). There is a possibility that each mutation mediates an antigenic variation of this virus. In the present study, no isolates lost reactivity with the polyclonal antibodies against C/Ann Arbor/1/50. A more than four-fold decrease in reactivity with the polyclonal antibodies was observed for the escape mutant possessing the E193K mutation. Among the natural isolates, lysine at position 193 was recognized in C/Paris/1/67 (C/Taylor lineage) and C/Great Lakes/1167/54 (C/Yamagata lineage) (Figure 3), but these strains did not show decreased reactivity with the polyclonal antibodies, and the E193K mutation was not inherited in further strains. Therefore, we could not identify E193K as a mutation responsible for antigenic drift. The selection of antigenic drift variants is thought to be a sequential event involving the stepwise accumulation of mutations through different individuals [32]. The frequency of variant selection in the presence of anti-HE MAbs was low; the frequencies ranged from 10^−4.62^ to 10^−7.58^ for the C/Ann Arbor/1/50 virus (Table 1) and 10^−7.11^ to 10^−9.25^ for the C/Yamagata/15/2004 virus (Table 2), which were lower than the frequencies of 10^−2.97^ to 10^−4.84^ for the A(H1N1)pdm virus [24]. Therefore, antigenic drift, which occurs as a result of point mutations in several antigenic sites, might be a rare or non-occurring event for the influenza C virus.

In the present study, we evaluated the epitopes of nine MAbs on the 3D structure of the HE protein. The data presented here are useful as a basis for future studies of the immunological and epidemiological characteristics of influenza C virus. Continued surveillance and antigenic analysis using the panel of MAbs characterized here would help determine the succeeding replacement of antigenic lineages or the possible emergence of a novel lineage of influenza C virus.

## Figures and Tables

**Table 1:** Characterization of anti-C/Ann Arbor/1/50 hemagglutinin-esterase (HE) monoclonal antibodies (MAbs) and selection frequencies of escape mutants of the C/Ann Arbor/1/50 virus.

		Antibody Titer			Selection Frequency (−log_10_)		
MAb	Isotype ^1^	ELISA (×10^4^) ^1^	HI	NT_50_^1^	CJP1	CJP3	CJP5
J9	IgG1	1600	128,000	160,000	6.18	6.53	6.69
U9	IgG1	200	128,000	200	5.87	5.30	6.10
Q5	IgG2a	1600	64,000	16,000	5.82	6.37	6.13
J14	IgG1	1000	256,000	8,000,000	7.16	6.28	6.97
K16	IgG1	640	80	320,000	5.58	4.62	5.05
U1	IgG1	200	16,000	20,000	6.96	7.58	6.36
U2	IgG1	100	6400	4000	5.92	5.80	5.94
D37	IgG2a	200	160	8000	― ^2^	―	―

^1^ Data are from our previous report [17]. ^2^ ―: not obtained.

**Table 2:** Characterization of anti-C/Yamagata/15/2004 HE MAbs and selection frequencies of escape mutants of the C/Yamagata/15/2004 virus.

		Antibody Titer			Selection Frequency (−log_10_)		
MAb	Isotype	ELISA (×10^4^)	HI	NT_50_	CYP1	CYP2	CYP4
YA3	IgG1	800	25,600	80,000	8.42	8.22	9.25
YA5	IgG1	800	12,800	40,000	8.17	7.11	8.19

**Table 3:** Amino acid mutations in the HE protein of escape mutants of the C/Ann Arbor/1/50 virus, their isolation efficiencies, and their effects on reactivity with each MAbs and chicken antiserum.

Amino Acid Change of Escape Mutants	MAb(s) Used for Selection of Escape Mutants	No. of Escape Mutants Derived from Parent Virus				Hemagglutination Inhibition Titer of MAb ^1^ or of Chicken Antiserum							
						MAb against C/Ann Arbor/1/50 Virus at Site:							Chicken Antiserum against C/Ann Arbor/1/50
						A-1				A-2	A-3		
		CJP1	CJP3	CJP5	Total	J9	U9	Q5	J14	K16	U1	U2	
None (Parent Virus)						128,000	128,000	64,000	256,000	80	16,000	6400	1280
N125D	J9		1	1	2	<20	―	―	3200	―	―	―	2560
K172R	J9	1	1	2	4	160	―	3200	―	―	―	―	2560
K172Q	J9	7	1	1	9	<20	―	640	―	―	―	―	2560
K172N	J9			2	2	<20	―	160	―	―	―	―	5120
N173I	J9	2	6	4	12	320	―	―	―	―	―	―	2560
N175S	J9, J14	9	2	3	14	<20	―	―	<20	―	―	―	1280
S192L	U9	9	10	4	23	―	160	―	―	―	―	―	2560
E193K	U9, Q5	6	5	9	20	―	<20	<20	―	<20	―	―	320
K235R	U9, Q5	4	5	4	13	―	<20	<20	―	―	―	―	2560
K235I	U9, Q5, J14			3	3	―	<20	<20	<20	―	―	―	1280
K235E	J14		3		3	―	<20	<20	<20	―	―	―	1280
D269N	J14		5	6	11	―	―	―	<20	<20	―	―	640
A65D	K16		1		1	―	―	―	―	<20	―	―	2560
R68W	K16		3		3	―	―	―	―	<20	―	―	2560
A351V	K16			7	7	―	―	―	―	<20	―	―	1280
D353G	K16	2		2	4	―	―	―	―	<20	―	―	1280
D353N	K16	3	1	1	5	―	―	―	―	<20	―	―	1280
D353Y	K16	1	2		2	―	―	―	―	<20	―	―	1280
L164P	U1, U2	4	10	2	16	―	―	―	―	―	160	<20	2560
L164S	U2	1			1	―	―	―	―	―	―	―	2560
K198E	U1, U2	8	10	11	29	―	―	―	―	―	320	20	1280
L201H	U1, U2	1		1	2	―	―	―	―	―	640	80	2560
L201I	U2	1			1	―	―	―	―	―	―	―	2560
L201R	U2			1	1	―	―	―	―	―	80	<20	1280
F203S	U1, U2	4		5	9	―	―	―	―	―	1280	160	1280
Total no. of mutants		63	66	69	198								

^1^ The hemagglutination inhibition (HI) titer is expressed as the reciprocal of the highest antibody dilution that completely inhibited hemagglutination. ―, a less than 10-fold lower or higher HI titer than that of the parent virus. 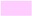 and 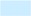 denote a greater than 10-fold lower HI titer of MAbs at sites A-1 and A-3 than that of the parent virus, respectively. 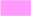, 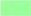, and 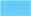 denote undetermined (less than 20) HI titer of MAbs at sites A-1, A-2, and A-3, respectively.

**Table 4:** Amino acid mutations in the HE protein of escape mutants of the C/Yamagata/15/2004 virus, their isolation efficiencies, and their effects on reactivity with each MAbs and chicken antiserum.

Amino Acid Change of Escape Mutants	MAb(s) Used for Selection of Escape Mutants	No. of Escape Mutants Derived from Parent Virus				Hemagglutination Inhibition Titer of MAb ^1^ or of Chicken Antiserum							
						Mab against C/Yamagata/15/2004 Virus at Site:		MAb against C/Ann Arbor/1/50 Virus at Site:					Chicken Antiserum against C/Yamagata/10/89 ^2^
						Y-1		A-1			A-3		
		CYP1	CYP2	CYP4	Total	YA3	YA5	U9	Q5	J14	U1	U2	
None (parent virus)						12,800	12,800	1280	12,800	32,000	6400	640	5120
K172R	YA5			1	1	―	―	―	<20	―	―	―	5120
K190N	YA3, YA5	2	8	5	15	―	―	80	―	―	―	―	5120
E193K	YA3, YA5	5	3	3	11	1280	640	<20	<20	320	―	―	640
192-195 deletion	YA3, YA5	9	7		16	<20	<20	<20	―	―	―	―	2560
198 deletion	YA3		1	4	5	<20	<20	<20	80	―	―	―	640
Total no. of mutants		16	19	13	48								

^1^ The HI titer is expressed as the reciprocal of the highest antibody dilution that completely inhibited hemagglutination. ― denotes a less than 10-fold lower or higher HI titer than that of the parent virus. 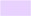 and 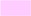 denote a greater than 10-fold lower HI titer of MAbs at sites Y-1 and A-1 than that of the parent virus, respectively. 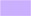 and 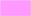 denote undetermined (less than 20) HI titer of MAbs at sites Y-1 and A-1, respectively. ^2^ The HE antigenicity of C/Yamagata/10/89 is identical to that of C/Yamagata/26/81 [10], which is a reference strain of the C/Yamagata lineage.

**Table 5:** Antigenic analysis of influenza C virus strains isolated from 1947 to 2016 by hemagglutination inhibition (HI) tests.

HE Lineage	Strain	HI Titer									
		MAbs of Respective Antigenic Site								Chicken Antiserum against:	
		A-1				A-3		Y-1			
		J9	U9	Q5	J14	U1	U2	YA3	YA5	C/Ann Arbor/1/50	C/Yamagata/10/89 ^1^
C/Taylor	C/Taylor/1233/47	64,000	64,000	32,000	256,000	16,000	3200	<^2^	<	1280	640
	C/Ann Arbor/1/50	64,000	64,000	16,000	25,6000	16,000	6400	<	<	1280	640
	C/Paris/1/67	512,000	<	<	512,000	16,000	3200	<	<	1280	320
C/Mississippi	C/Greece/1/79	<	<	<	256,000	32,000	6400	<	<	320	160
	C/Mississippi/80	<	<	<	256,000	32,000	12,800	<	<	160	160
	C/Yamagata/26/2004	<	<	<	256,000	32,000	12,800	<	<	160	160
C/Aichi	C/Johannesburg/1/66	<	16,000	<	512,000	16,000	3200	<	<	160	320
	C/Georgia/1/69	<	16,000	<	512,000	<	20	<	<	160	320
	C/Aichi/1/81	<	16,000	<	256,000	640	80	<	<	80	320
	C/Yamagata/4/92	<	16,000	<	256,000	640	80	<	<	160	320
C/Yamagata	C/Great Lakes/1167/54	6400	3200	320	16,000	<	20	<	<	1280	2560
	C/Sapporo/71	640	640	1600	128,000	3200	320	6400	6400	160	1280
	C/Yamagata/26/81	<	640	1600	64,000	40	160	12,800	12,800	320	2560
	C/Miyagi/2/92	<	640	320	16,000	32,000	12,800	25,600	25,600	320	2560
	C/Yamagata/15/2004	<	640	1600	32,000	20	80	12,800	12,800	320	2560
C/Kanagawa	C/Yamagata/64	128,000	1280	32,000	256,000	<	20	3200	3200	320	320
	C/Aomori/74	<	160	320	128,000	3200	640	1600	800	320	640
	C/Kanagawa/1/76	<	40	320	128,000	<	<	<	<	160	160
	C/Miyagi/77	<	16,000	320	256,000	<	<	<	<	160	320
	C/Miyagi/9/96	<	40	80	16,000	<	<	<	<	160	640
	C/Yamagata/13/2014	<	320	160	256,000	<	<	160	80	160	320
C/Sao Paulo	C/Sao Paulo/378/82	<	16,000	800	64,000	32,000	12,800	12,800	6400	640	320
	C/Fukuoka/1/2005	<	32,000	1600	128,000	16,000	3200	6400	6400	320	320
	C/Yamagata/33/2014	<	32,000	1600	32,000	16,000	3200	6400	3200	640	640
	C/Yamagata/30/2014	1280	32,000	800	64,000	8000	1600	<	<	320	320
	C/Yamagata/1/2016	128,000	32,000	1600	128,000	32,000	12,800	<	<	320	320

^1^ The HE antigenicity of C/Yamagata/10/89 is identical to that of C/Yamagata/26/81 [10]. ^2^ < denotes less than 20.

## Supplementary Materials

The following are available online at http://www.mdpi.com/1999-4915/10/8/417/s1: Table S1: Background information of the hemagglutinin-esterase gene sequences used in this study, and Figure S1: Monthly distribution of influenza C viruses in Japan between 1964 and 2016.

## References

[1] Pfeifer J.B., Compans R.W. (1984). Structure of the influenza C glycoprotein gene as determined from cloned DNA. Virus Res..

[2] Vlasak R., Krystal M., Nacht M., Palese P. (1987). The influenza C virus glycoprotein (HE) exhibits receptor-binding (hemagglutinin) and receptor-destroying (esterase) activities. Virology.

[3] Herrler G., Dürkop I., Becht H., Klenk H.D. (1988). The glycoprotein of influenza C virus is the haemagglutinin, esterase and fusion factor. J. Gen. Virol..

[4] Formanowski F., Meier-Ewert H. (1988). Isolation of the influenza C virus glycoprotein in a soluble form by bromelain digestion. Virus Res..

[5] Kimura H., Abiko C., Peng G., Muraki Y., Sugawara K., Hongo S., Kitame F., Mizuta K., Numazaki Y., Suzuki H. (1997). Interspecies transmission of influenza C virus between humans and pigs. Virus Res..

[6] Katagiri S., Ohizumi A., Homma M. (1983). An outbreak of type C influenza in a children’s home. J. Infect. Dis..

[7] Matsuzaki Y., Katsushima N., Nagai Y., Shoji M., Itagaki T., Sakamoto M., Kitaoka S., Mizuta K., Nishimura H. (2006). Clinical features of influenza C virus infection in children. J. Infect. Dis..

[8] Thielen B.K., Friedlander H., Bistodeau S., Shu B., Lynch B., Martin K., Bye E., Como-Sabetti K., Boxrud D., Strain A.K. (2018). Detection of influenza C viruses among outpatients and patients hospitalized for severe acute respiratory infection, Minnesota, 2013–2016. Clin. Infect. Dis..

[9] Matsuzaki Y., Sugawara K., Furuse Y., Shimotai Y., Hongo S., Oshitani H., Mizuta K., Nishimura H. (2016). Genetic lineage and reassortment of influenza C viruses circulating between 1947 and 2014. J. Virol..

[10] Matsuzaki Y., Muraki Y., Sugawara K., Hongo S., Nishimura H., Kitame F., Katsushima N., Numazaki Y., Nakamura K. (1994). Cocirculation of two distinct groups of influenza C virus in Yamagata City, Japan. Virology.

[11] Matsuzaki Y., Mizuta K., Sugawara K., Tsuchiya E., Muraki Y., Hongo S., Suzuki H., Nishimura H. (2003). Frequent reassortment among influenza C viruses. J. Virol..

[12] Matsuzaki Y., Sugawara K., Abiko C., Ikeda T., Aoki Y., Mizuta K., Katsushima N., Katsushima F., Katsushima Y., Itagaki T. (2014). Epidemiological information regarding the periodic epidemics of influenza C virus in Japan (1996–2013) and the seroprevalence of antibodies to different antigenic groups. J. Clin. Virol..

[13] Matsuzaki M., Sugawara K., Adachi K., Hongo S., Nishimura H., Kitame F., Nakamura K. (1992). Location of neutralizing epitopes on the hemagglutinin-esterase protein of influenza C virus. Virology.

[14] Rosenthal P.B., Zhang X., Formanowski F., Fitz W., Wong C.H., Meier-Ewert H., Skehel J.J., Wiley D.C. (1998). Structure of the haemagglutinin-esterase-fusion glycoprotein of influenza C virus. Nature.

[15] Sugawara K., Nishimura H., Kitame F., Nakamura K. (1986). Antigenic variation among human strains of influenza C virus detected with monoclonal antibodies to gp88 glycoprotein. Virus Res..

[16] Sugawara K., Kitame F., Nishimura H., Nakamura K. (1988). Operational and topological analyses of antigenic sites on influenza C virus glycoprotein and their dependence on glycosylation. J. Gen. Virol..

[17] Sugawara K., Nishimura H., Hongo S., Muraki Y., Kitame F., Nakamura K. (1993). Construction of an antigenic map of the haemagglutinin-esterase protein of influenza C virus. J. Gen. Virol..

[18] Matsuzaki Y., Abiko C., Mizuta K., Sugawara K., Takashita E., Muraki Y., Suzuki H., Mikawa M., Shimada S., Sato K. (2007). A nationwide epidemic of influenza C virus infection in Japan in 2004. J. Clin. Microbiol..

[19] Matsuzaki Y., Mizuta K., Kimura H., Sugawara K., Tsuchiya E., Suzuki H., Hongo S., Nakamura K. (2000). Characterization of antigenically unique influenza C virus strains isolated in Yamagata and Sendai cities, Japan, during 1992–1993. J. Gen. Virol..

[20] Tanaka S., Aoki Y., Matoba Y., Yahagi K., Mizuta K., Itagaki T., Katsushima F., Katsushima Y., Matsuzaki Y. (2015). The dominant antigenic group of influenza C infections changed from C/Sao Paulo/378/82-lineage to C/Kanagawa/1/76-lineage in Yamagata, Japan, in 2014. Jpn. J. Infect. Dis..

[21] Hongo S., Sugawara K., Homma M., Nakamura K. (1986). The functions of oligosaccharide chains associated with influenza C viral glycoproteins. II. The role of carbohydrates in the antigenic properties of influenza C viral glycoproteins. Arch. Virol..

[22] Kawamura H., Tashiro M., Kitame F., Homma M., Nakamura K. (1986). Genetic variation among human strains of influenza C virus isolated in Japan. Virus Res..

[23] Furuse Y., Matsuzaki Y., Nishimura H., Oshitani H. (2016). Analyses of evolutionary characteristics of the hemagglutinin-esterase gene of influenza C virus during a period of 68 years reveals evolutionary patterns different from influenza A and B viruses. Viruses.

[24] Matsuzaki Y., Sugawara K., Nakauchi M., Takahashi Y., Onodera T., Tsunetsugu-Yokota Y., Matsumura T., Ato M., Kobayashi K., Shimotai Y. (2014). Epitope mapping of the hemagglutinin molecule of A/(H1N1)pdm09 influenza virus by using monoclonal antibody escape mutants. J. Virol..

[25] Tsuchiya E., Sugawara K., Hongo S., Matsuzaki Y., Muraki Y., Li Z.N., Nakamura K. (2001). Antigenic structure of the haemagglutinin of human influenza A/H2N2 virus. J. Gen. Virol..

[26] Tamura K., Stecher G., Peterson D., Filipski A., Kumar S. (2013). MEGA6: Molecular evolutionary genetics analysis version 6.0. Mol. Biol. Evol..

[27] Sugahara K., Hongo S., Sugawara K., Li Z.N., Tsuchiya E., Muraki Y., Matsuzaki Y., Nakamura K. (2001). Role of individual oligosaccharide chains in antigenic properties, intracellular transport, and biological activities of influenza C virus hemagglutinin-esterase protein. Virology.

[28] Adachi K., Kitame F., Sugawara K., Nishimura H., Nakamura K. (1989). Antigenic and genetic characterization of three influenza C strains isolated in the Kinki district of Japan in 1982–1983. Virology.

[29] Ohyama S., Adachi K., Sugawara K., Hongo S., Nishimura H., Kitame F., Nakamura K. (1992). Antigenic and genetic analyses of eight influenza C strains isolated in various areas of Japan during 1985–1989. Epidemiol. Infect..

[30] Matsuzaki Y., Takao S., Shimada S., Mizuta K., Sugawara K., Takashita E., Muraki Y., Hongo S., Nishimura H. (2004). Characterization of antigenically and genetically similar influenza C viruses isolated in Japan during the 1999–2000 season. Epidemiol. Infect..

[31] Matsuzaki Y., Sugawara K., Mizuta K., Tsuchiya E., Muraki Y., Hongo S., Suzuki H., Nakamura K. (2002). Antigenic and genetic characterization of influenza C viruses which caused two outbreaks in Yamagata City, Japan, in 1996 and 1998. J. Clin. Microbiol..

[32] Wright P.F., Neumann G., Kawaoka Y., Knipe D.M., Howley P.M. (2013). Orthomyxoviruses. Fields Virology.

[33] Air G.M. (2012). Influenza neuraminidase. Influenza Other Respir. Viruses.

[34] Hachinohe S., Sugawara K., Nishimura H., Kitame F., Nakamura K. (1989). Effect of anti-haemagglutinin-esterase glycoprotein monoclonal antibodies on the receptor-destroying activity of influenza C virus. J. Gen. Virol..

[35] Umetsu Y., Sugawara K., Nishimura H., Hongo S., Matsuzaki M., Kitame F., Nakamura K. (1992). Selection of antigenically distinct variants of influenza C viruses by the host cell. Virology.

