# Supplementary material for: Neutralizing Epitopes and Residues Mediating the Potential Antigenic Drift of the Hemagglutinin-Esterase Protein of Influenza C Virus
Source: Viruses. 2018 Aug 9;10(8):417. doi: 10.3390/v10080417 (PMC6116000; doi:10.3390/v10080417)
Supplement: Supplementary file 1 [file viruses-10-00417-s001.zip › Figure S1.pdf]

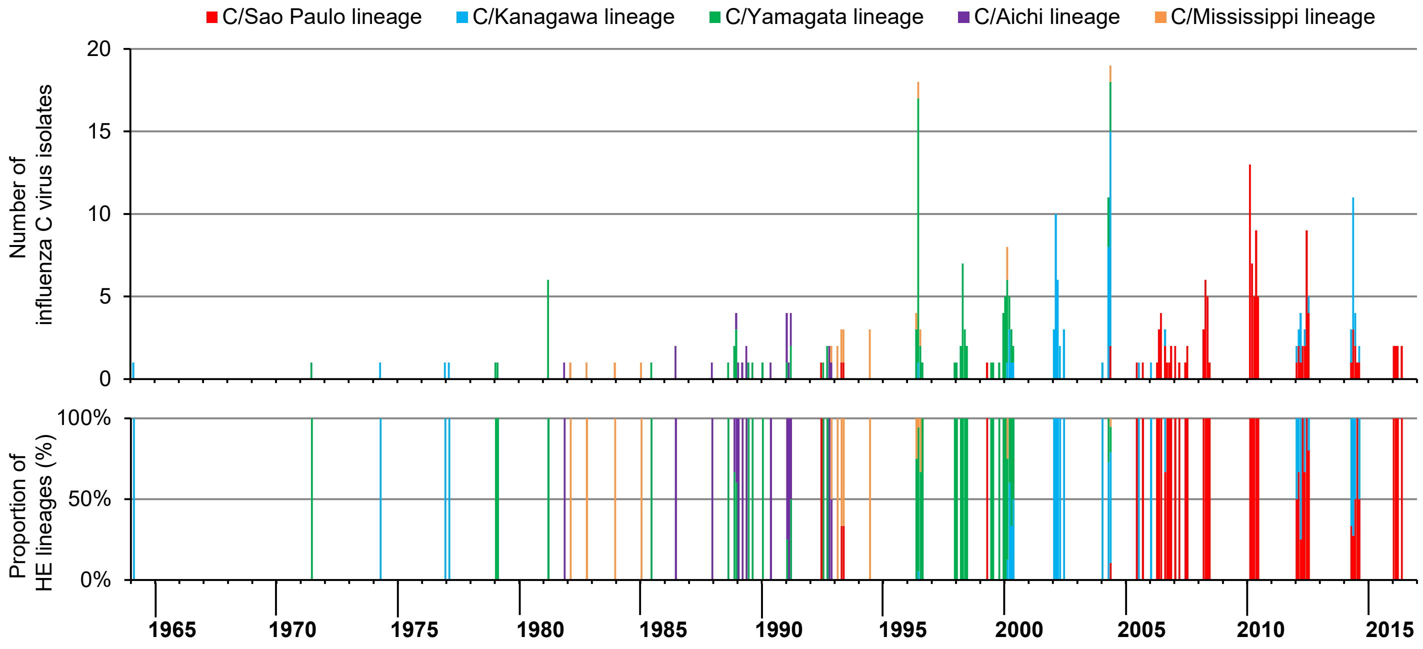

Figure S1: Monthly distribution of influenza C viruses in Japan between 1964 and 2016.

The number of influenza C virus isolates was obtained from our previous reports: Matsuzaki et al. [10-12,19,30,31], Kimura et al. [5], Tanaka, et al [20], Kawamura et al. [22], Adachi et al. [28], and Ohyama et al. [29].
