# Supplementary material for: Neutralizing Epitopes and Residues Mediating the Potential Antigenic Drift of the Hemagglutinin-Esterase Protein of Influenza C Virus
Source: Viruses. 2018 Aug 9;10(8):417. doi: 10.3390/v10080417 (PMC6116000; doi:10.3390/v10080417)
Supplement: Supplementary file 1 [file viruses-10-00417-s001.zip › Table S1.doc]

Table S1: Background information of the hemagglutinin-esterase gene sequences used in this study.

| **Strain** | **Accession no.** | **Collection_date** | **HE lineage** | **Reference** |
| --- | --- | --- | --- | --- |
| C/Taylor/1233/47 | LC122632 | 1947 | C/Taylor | [9] |
| C/Ann Arbor/1/50 | AB126194 | 1950 | C/Taylor | [9] |
| C/Great Lakes/1167/54 | LC122633 | 1954 | C/Yamagata | [9] |
| C/Yamagata/64 | D63467 | 1964 | C/Kanagawa | [9] |
| C/Johannesburg/1/66 | M17868 | 1966 | C/Aichi | [9] |
| C/Johannesburg/4/67 | AB035358 | 1967 | C/Aichi | [9] |
| C/Paris/1/67 | AB035357 | 1967 | C/Taylor | [9] |
| C/Georgia/1/69 | AB035359 | 1969 | C/Aichi | [9] |
| C/Sapporo/71 | D63468 | 1971 | C/Yamagata | [9] |
| C/Aomori/74 | D63469 | 1974 | C/Kanagawa | [9] |
| C/New Jersey/1/76 | AB035362 | 1976 | C/Yamagata | [9] |
| C/Kanagawa/1/76 | D63470 | 1976 | C/Kanagawa | [9] |
| C/Miyagi/77 | D63471 | 1977 | C/Kanagawa | [9] |
| C/California/78 | LC122634 | 1978 | C/Yamagata | [9] |
| C/Kansas/1/79 | AB035360 | 1979 | C/Aichi | [9] |
| C/Greece/1/79 | AB035363 | 1979 | C/Mississippi | [9] |
| C/Kyoto/1/79 | D63472 | 1979 | C/Yamagata | [9] |
| C/Shizuoka/79 | AB086656 | 1979 | C/Yamagata | [9] |
| C/Mississippi/80 | LC122635 | 1980 | C/Mississippi | [9] |
| C/Aichi/1/81 | D28970 | 1981 | C/Aichi | [9] |
| C/Yamagata/7/81 | AB899775 | 1981-03-18 | C/Yamagata | [9] |
| C/Yamagata/10/81 | AB899776 | 1981-03-18 | C/Yamagata | [9] |
| C/Yamagata/26/81 | D28971 | 1981-03-18 | C/Yamagata | [9] |
| C/pig/Beijing/115/81 | LC122636 | 1981 | C/Yamagata | [9] |
| C/Sao Paulo/378/82 | AB035364 | 1982 | C/Sao Paulo | [9] |
| C/Nara/82 | M25362 | 1982 | C/Mississippi | [9] |
| C/England/892/83 | LC122637 | 1983 | C/Yamagata | [9] |
| C/Yamagata/7/88 | D28967 | 1988-11-03 | C/Yamagata | [9] |
| C/Yamagata/9/88 | D28969 | 1988-12-12 | C/Aichi | [9] |
| C/Yamagata/10/89 | D49361 | 1989-08-03 | C/Yamagata | [9] |
| C/Miyagi/5/91 | AB086659 | 1991-02-25 | C/Aichi | [9] |
| C/Miyagi/9/91 | D87394 | 1991-03-27 | C/Yamagata | [5] |
| C/Miyagi/2/92 | D87392 | 1991-09-27 | C/Yamagata | [5] |
| C/Yamagata/4/92 | AB899777 | 1992-11-19 | C/Aichi | [9] |
| C/Yamagata/5/92 | D78433 | 1992-11-26 | C/Mississippi | [9] |
| C/Yamagata/1/93 | AB035365 | 1993-04-30 | C/Sao Paulo | [9] |
| C/Miyagi/1/93 | AB064407 | 1993-02-02 | C/Mississippi | [9] |
| C/Miyagi/3/93 | AB064408 | 1993-04-13 | C/Mississippi | [9] |
| C/Miyagi/7/93 | AB064411 | 1993-05-19 | C/Mississippi | [9] |
| C/Miyagi/8/96 | AB064406 | 1996-05-23 | C/Mississippi | [9] |
| C/Miyagi/9/96 | AB086664 | 1996-06-03 | C/Kanagawa | [9] |
| C/Yamagata/8/96 | AB064398 | 1996-06-13 | C/Yamagata | [9] |
| C/Yamagata/13/98 | AB064403 | 1998-06-08 | C/Yamagata | [9] |
| C/Aichi/1/99 | AB182357 | 1999-04-07 | C/Sao Paulo | [9] |
| C/Yamagata/3/2000 | AB099413 | 2000-02-02 | C/Mississippi | [9] |
| C/Yamagata/6/2000 | AB099414 | 2000-03-21 | C/Yamagata | [9] |
| C/Yamagata/5/2002 | LC122638 | 2002-02-07 | C/Kanagawa | [9] |
| C/Yamagata/7/2002 | AB899778 | 2002-02-16 | C/Kanagawa | [9] |
| C/Miyagi/4/2002 | LC122639 | 2002-01-24 | C/Kanagawa | [9] |
| C/Miyagi/11/2002 | LC122640 | 2002-02-22 | C/Kanagawa | [9] |
| C/Miyagi/20/2002 | LC122641 | 2002-03-17 | C/Yamagata | [9] |
| C/Miyagi/28/2002 | LC122642 | 2002-04-22 | C/Kanagawa | [9] |
| C/Miyagi/31/2002 | LC122643 | 2002-06-18 | C/Kanagawa | [9] |
| C/Yamagata/3/2004 | AB252153 | 2004-04-16 | C/Kanagawa | [9] |
| C/Yamagata/14/2004 | AB899779 | 2004-05-07 | C/Kanagawa | [9] |
| C/Yamagata/15/2004 | AB252154 | 2004-05-11 | C/Yamagata | [9] |
| C/Yamagata/18/2004 | LC122644 | 2004-05-14 | C/Sao Paulo | [9] |
| C/Yamagata/26/2004 | LC122645 | 2004-05-28 | C/Mississippi | [9] |
| C/Yamagata/27/2004 | AB252155 | 2004-05-24 | C/Sao Paulo | [9] |
| C/Miyagi/12/2004 | AB252156 | 2004-02-27 | C/Kanagawa | [9] |
| C/Miyagi/25/2004 | LC122646 | 2004-03-31 | C/Kanagawa | [9] |
| C/Miyagi/42/2004 | AB252157 | 2004-05-27 | C/Kanagawa | [9] |
| C/Miyagi/47/2004 | LC122647 | 2004-06-18 | C/Kanagawa | [9] |
| C/Yamagata/1/2005 | LC122648 | 2005-06-07 | C/Sao Paulo | [9] |
| C/Yamagata/2/2005 | LC122649 | 2005-07-05 | C/Kanagawa | [9] |
| C/Yamagata/3/2005 | LC122650 | 2005-09-07 | C/Sao Paulo | [9] |
| C/Miyagi/2/2005 | LC122651 | 2005-04-07 | C/Kanagawa | [9] |
| C/Miyagi/4/2005 | LC122652 | 2005-05-16 | C/Kanagawa | [9] |
| C/Fukuoka/1/2005 | LC122653 | 2005-12-28 | C/Sao Paulo | [9] |
| C/Fukuoka/2/2006 | LC122654 | 2006-03-08 | C/Kanagawa | [9] |
| C/Yamagata/1/2006 | LC122655 | 2006-01-23 | C/Kanagawa | [9] |
| C/Yamagata/9/2006 | LC122656 | 2006-06-13 | C/Sao Paulo | [9] |
| C/Yamagata/13/2006 | LC122657 | 2006-08-08 | C/Kanagawa | [9] |
| C/Yamagata/14/2006 | LC013262 | 2006-08-28 | C/Sao Paulo | [9] |
| C/Yamagata/20/2006 | LC122658 | 2006-11-24 | C/Sao Paulo | [9] |
| C/Miyagi/4/2006 | LC122659 | 2006-06-04 | C/Sao Paulo | [9] |
| C/Yamagata/1/2007 | LC122660 | 2007-01-17 | C/Sao Paulo | [9] |
| C/Yamagata/3/2008 | LC122661 | 2008-03-26 | C/Sao Paulo | [9] |
| C/Yamagata/11/2008 | LC122662 | 2008-04-30 | C/Sao Paulo | [9] |
| C/Yamagata/16/2008 | LC122663 | 2008-05-19 | C/Sao Paulo | [9] |
| C/Yamagata/18/2008 | LC013263 | 2008-06-25 | C/Sao Paulo | [9] |
| C/Miyagi/2/2008 | LC122664 | 2008-02-21 | C/Sao Paulo | [9] |
| C/Yamagata/2/2010 | AB899780 | 2010-02-09 | C/Sao Paulo | [9] |
| C/Miyagi/3/2010 | LC122665 | 2010-04-12 | C/Sao Paulo | [9] |
| C/Tokyo/3/2010 | LC122666 | 2010-04-23 | C/Sao Paulo | [9] |
| C/Victoria/2/2012 | KM504280 | 2012 | C/Sao Paulo | [9] |
| C/Yamagata/1/2012 | LC122667 | 2012-01-16 | C/Kanagawa | [9] |
| C/Yamagata/3/2012 | LC122668 | 2012-02-07 | C/Sao Paulo | [9] |
| C/Yamagata/4/2012 | AB899781 | 2012-02-22 | C/Kanagawa | [9] |
| C/Yamagata/7/2012 | LC122669 | 2012-03-14 | C/Kanagawa | [9] |
| C/Yamagata/14/2012 | LC122670 | 2012-06-01 | C/Sao Paulo | [9] |
| C/Yamagata/28/2012 | LC122671 | 2012-07-12 | C/Kanagawa | [9] |
| C/Yamagata/29/2012 | AB899782 | 2012-07-30 | C/Sao Paulo | [9] |
| C/Miyagi/2/2012 | LC122672 | 2012-01-26 | C/Sao Paulo | [9] |
| C/Fukuoka/1/2012 | LC122673 | 2012-04-11 | C/Sao Paulo | [9] |
| C/Yamagata/6/2014 | LC013253 | 2014-04-16 | C/Sao Paulo | [9] |
| C/Yamagata/13/2014 | LC013254 | 2014-05-14 | C/Kanagawa | [9] |
| C/Yamagata/16/2014 | LC013257 | 2014-05-21 | C/Kanagawa | [9] |
| C/Yamagata/30/2014 | LC013258 | 2014-06-04 | C/Sao Paulo | [9] |
| C/Yamagata/32/2014 | LC013259 | 2014-06-17 | C/Sao Paulo | [9] |
| C/Yamagata/33/2014 | LC013260 | 2014-07-22 | C/Sao Paulo | [9] |
| C/Yamagata/35/2014 | LC013261 | 2014-08-20 | C/Kanagawa | [9] |
| C/Miyagi/2/2014 | LC122674 | 2014-04-30 | C/Kanagawa | [9] |
| C/Miyagi/5/2014 | LC122675 | 2014-05-17 | C/Sao Paulo | [9] |
| C/Miyagi/6/2014 | LC122676 | 2014-06-02 | C/Sao Paulo | [9] |
| C/Tokyo/1/2014 | LC122677 | 2014-02-01 | C/Sao Paulo | [9] |
| C/Tokyo/4/2014 | LC122678 | 2014-04-23 | C/Sao Paulo | [9] |
| C/Yamagata/1/2016 | LC385860 | 2016-01-18 | C/Sao Paulo | This study |
| C/Yamagata/4/2016 | LC385861 | 2016-01-25 | C/Sao Paulo | This study |
| C/Yamagata/6/2016 | LC385862 | 2016-02-02 | C/Sao Paulo | This study |
| C/Yamagata/12/2016 | LC385863 | 2016-02-08 | C/Sao Paulo | This study |
| C/Yamagata/24/2016 | LC385864 | 2016-03-26 | C/Sao Paulo | This study |
| C/Yamagata/26/2016 | LC385865 | 2016-03-28 | C/Sao Paulo | This study |
| C/Yamagata/35/2016 | LC385866 | 2016-05-11 | C/Sao Paulo | This study |
| C/Yamagata/36/2016 | LC385867 | 2016-05-13 | C/Sao Paulo | This study |
